# Adolescent HIV—Cause for Concern in Southern Africa

**DOI:** 10.1371/journal.pmed.1000227

**Published:** 2010-02-02

**Authors:** Glenda E. Gray

**Affiliations:** Perinatal HIV Research Unit, University of the Witwatersrand, Johannesburg, South Africa

## Abstract

Glenda Gray discusses the implications of a new study that found that almost half of all adolescents hospitalized in Zimbabwe were HIV-infected.

**Linked Research Article**

This Perspective discusses the following new study published in *PLoS Medicine*:

Ferrand R, Bandason T, Musvaire, P, Larke N, Nathoo K, et al. (2010) Causes of Acute Hospitalization in Adolescence: Burden and Spectrum of HIV-Related Morbidity in a Country with an Early-Onset and Severe HIV Epidemic. PLoS Med 7(2): e1000178. doi:10.1371/journal.pmed.1000178

In 2006, the Society for Adolescent Medicine issued its second position paper on HIV/AIDS in adolescents [1]. It noted that although great progress had been made in the scientific understanding, diagnosis and treatment of HIV, and the prevention of perinatal transmission, there was a growing HIV crisis in the developing world. At least half of all new infections in the developing world were amongst youth and young adults, and a substantial number of teenagers and young adults were already living with HIV/AIDS [2].

As HIV epidemics mature, increasing numbers of children infected perinatally survive and will present with HIV-related symptoms in older childhood and adolescence. Whilst the epidemiology of sexually acquired HIV infection amongst 15–24 year olds is well described in southern Africa [3]–[5], few data on the prevalence and disease pattern of perinatally acquired HIV infection in older children and adolescence exist. Recent data from a household survey conducted in South Africa in 2008 estimated the prevalence of HIV in children aged 2–14 years to be 2.5% (95% confidence interval 1.9–3.5) [6]. The survey indicates the relatively high prevalence of HIV in children and adolescents in this region. Most of these infections are acquired early in life and are probably undiagnosed.

## The Survival of Infected Children

Little is also known about the survival of HIV-infected children in Africa beyond 5 years of age. Some studies have estimated that 38% of children will be slow progressors and that the estimated cumulated mortality at 15 years will be 83% [7]. Others estimate a 67% survival at 1 year, 39% at 5 years, and 13% survival at 10 years [8]. Decreased survival of HIV-infected children in Africa to date is attributed to lack of access to antiretroviral therapy (ART), delayed diagnosis, and inadequate management due to lack of expertise and resources.

There are few data on the impact that HIV has on ill-health, morbidity, and mortality in adolescence in southern Africa. A recent study modeled demographic, HIV prevalence, mother-to-child transmission, and child survival data in South Africa and Zimbabwe [9]. It estimated that without treatment, the HIV prevalence among 10 year olds in South Africa will increase from 2.1% in 2008, to 3.3% in 2020, whereas in Zimbabwe it will decrease from 3.2% in 2008 to 1.6% in 2020. Deaths among untreated slow progressors will increase in South Africa from 7,000 per year in 2008 to 23,000 per year in 2030, and in Zimbabwe deaths will peak in 2014 at 9,700 per year, from 8,000 per year in 2008. The toll of adolescent HIV on other health outcomes such as hospital admissions in these settings has yet to be quantified.

## A New Study on Hospitalizations

In this issue of *Plos Medicine*, Ferrand and colleagues present data on the causes of acute hospitalization in adolescence in Zimbabwe [10]. HIV has become the single most common cause of acute admission and in-hospital death amongst adolescents in Harare. Almost half of all adolescents hospitalized were found to be HIV infected, and most of them were severely immunosuppressed, with the major route of transmission being attributed to perinatal transmission. Those admitted were more likely to be stunted, have pubertal delay, be a maternal orphan, or have an HIV-infected mother as compared to non-HIV admissions. Unlike their HIV-negative counterparts, who were largely admitted for trauma or an acute exacerbation of a chronic medical condition, HIV-infected adolescents were more likely to be admitted for an infection with tuberculosis, pneumonia, cryptococcosis, and septicemia. HIV-infected adolescents were also almost four times more likely to die. The risk of in-hospital death was increased in those HIV-infected adolescents who had underlying chronic complications.

These high rates of in-hospital mortality are also seen in other parts of the region. A study conducted in Zambia amongst HIV-antibody positive children aged 1–14 years found that a single hospitalization for a severe bacterial infection increased the risk of death by 42%, and a further hospitalization doubled this risk again [11]. Interventions to decrease HIV-related deaths that occur in hospitals require urgent investigation.

## Diagnosis, Clinical Manifestations, and Disease Outcome

Older children and adolescents are diagnosed late in Africa despite most guardians suspecting their children of being HIV infected before diagnosis [12]. The median age of diagnosis in some studies has been found to be between 11–12 years of age [10],[12], with a delay of 3.5 years (interquartile range, 1–6 years) between the first serious illness and diagnosis of HIV infection. In addition, when these adolescents are diagnosed, they are already below average for height and weight, have moderate to severe immunodeficiency, and have had recurrent infections as well as tuberculosis.

Most of the hospital admissions for HIV-infected adolescents in Harare in Ferrand et al. 's study were for infectious diseases [10]. It is unclear whether any data were collected on any underlying depression or other concomitant mental health diagnoses. Few data from southern Africa are available on the psychological manifestations, depressive symptoms, and psychiatric admissions for children and adolescents infected with HIV.

In contrast, studies from the developed world show high rates of admissions for psychiatric reasons amongst HIV-infected children and adolescents. In the United States, the PACTG 219C study—a prospective cohort study designed to examine long-term outcomes among HIV-infected children and HIV-exposed uninfected infants—found the incidence of psychiatric admissions to be 6.17 cases per 1,000 person-years of follow-up, which was significantly higher than that reported in the general population (1.70 cases per 1,000 person-years) [13]. In this study, the most common reasons for psychiatric hospitalization for HIV-infected children were for depression or behavioural disorders. The median age for first psychiatric admission was 11 years (range 4–17 years). Knowledge of HIV status increased the risk of hospital admissions 6-fold, and having experienced a significant life event increased the risk of hospitalization 3-fold. Almost half of the admitted children required multiple psychiatric admissions.

A previous study conducted amongst HIV-infected adolescents in Harare shows the tremendous burden HIV has on the family [12]. More than half of the adolescents participating in the study had lost both parents, and chronic ill health was reported in 44% of the surviving parents. Almost half of the adolescents were caring for sick parents, guardians, and/or siblings. The impact of death and chronic ill-health of a caregiver/and or siblings on the mental health of HIV-infected adolescents in southern Africa requires further description.

## Response to ART

Access to ART will improve health outcomes and long-term survival of any child that is infected early on in life, irrespective of setting. In the developed world, the long-term effects of protease-inhibitor–based combination therapy have shown greater improvements in CD4% in younger children as compared to older children. These findings may reflect greater thymic productivity in pre-adolescent children than in adolescents and adults [14] or other factors such as poorer treatment adherence in older children.

Kekitiinwa and colleagues examined the impact of ART across different geographical settings [15]. They looked at data describing HIV and early growth responses to ART across childhood, and compared initial responses to ART in the United Kingdom/Ireland and Uganda. They found that although early mortality after ART initiation was 3-fold higher in Uganda as compared to the United Kingdom/Ireland, older children and adolescents in Uganda had a superior virological response to ART compared with those from the United Kingdom/Ireland [15]. This difference was largely attributed to successful adolescent support programs at the Mulago Hospital in Kampala.

Additional data from Uganda, assessing the impact of ART on growth and sexual maturation in HIV-infected adolescents, showed appropriate virological and immunological responses to ART, as well as improvements in growth and to a much lesser extent, sexual maturation [16]. In a cohort of 118 perinatally infected, treatment naïve 10–19 year olds, the effect of antiretroviral was evaluated for a period of 12 months. At enrolment, the median CD4 count was 124, which had increased to 304 by 6 months, and to 370 by 12 months of treatment. ART was virologically suppressive in 79% of adolescents at 6 months, and by 89% at 12 months.

## Disclosing HIV Diagnosis to Adolescents

Disclosure issues abound both in the developed and developing world. However, studies suggest that there are medical benefits to disclosure of HIV infection status to children and adolescents. Children and adolescents who know their HIV status appear more likely to accept medical care and have a higher self-esteem as compared to youth that are unaware of their status [17],[18]. Nondisclosure can be associated with anxiety and depression, in addition to being excluded from social support. Reluctance of parents and caregivers to disclose the HIV status to a child or an adolescent is usually based on the fear of discrimination and stigma, toward both the adolescent and the family as a whole [19],[20].

The American Academy of Pediatrics strongly recommends the disclosure of HIV status to adolescents [21], so that they are fully informed about all aspects of their health, including their sexual behaviour. Ferrand and colleagues document a high rate of disclosure amongst HIV-infected adolescents in their study [10]. Nevertheless, they strongly recommend that health professionals include adolescents in routine provider-initiated testing and counseling, and assist guardians with disclosure as a way to improve early diagnosis and adherence to subsequent ART.

## Sexual Activity

Adolescents with moderate or severe immunosuppression are less likely to have adrenarche as compared to HIV-uninfected children of their own age [22]. However, the median age of sexual debut of adolescents who acquired their infection perinatally in southern Africa is unknown. In a study conducted in the US, amongst 40 HIV-positive adolescents/young adults, it was found that 28% of youth (mean age of 16.6 years), reported being sexually active [23]. When re-interviewed about 2 years later, 41% (mean age 18.3 years), were sexually active. Other studies examining sexual behaviour of adolescents infected with HIV as infants report sexually activity ranging from 18% (mean age of 15.5 years) [24], to 59% (mean age 18.5 years) in HIV-infected adolescents with hemophilia who ever reported prior sexual intercourse [25].

Of concern in these studies is that although self-efficacy around condom use was deemed to be high, it was not 100% guaranteed [23]. In various studies, between 63% to 80% of perinatally infected adolescents [24],[25] reported using condoms. HIV knowledge of sexual transmission has been found to be low [23], and these data highlight the need to provide risk-reduction counseling to adolescents who acquire HIV early in life.

## Conclusion

There is a substantial burden of HIV infection in adolescents in southern Africa who acquired HIV perinatally. It is evident that they contribute substantially to hospital admissions and in-hospital deaths. There is an urgent need for services that will be able to provide accessible and appropriate HIV testing, counseling, and support, as well as facilitate access to ART and appropriate sexual risk-reduction interventions. The adolescents admitted to hospitals in Harare could have benefited from early diagnosis and concomitant initiation of ART, and this absence of treatment should not continue to be the plight of similar adolescents in our region.

**Box 1. Five Key Papers in the Field of Adolescent HIV**

**Ferrand RA, Banson T, Musvaire P, Larke N, Nathoo K, et al., 2010** [**10**] **.** This paper demonstrates the burden of HIV infection in adolescents who acquired HIV infection in early life and how it contributes significantly to hospital admissions and in-hospital mortality. The article also highlights the need for early diagnosis, which will enable HIV-infected adolescents to benefit from earlier access to treatment and care.

**Shisana O, Rehle T, Simbayi LC, Zuma K, Jooste S,** **et al., 2009** [**6**] **.** This report on a national household survey conducted in South Africa shows the prevalence and incidence of HIV by age, gender, province, and geographic locality. The report also assesses risk behaviour, such as condom use, multiple concurrent partners, and sexual debut over time.

**Ferrand** **RA, Corbett EL, Wood R, Hargrove J, Ndhlovu CE,** **et al., 2009** [**9**] **.** This paper models the time course and magnitude of the AIDS epidemic among older children and adolescents in Southern Africa. These data are important for health policy makers and economists who need to evaluate the impact that the HIV epidemic has on health resources in Southern Africa.

**Walker** **AS, Mulenga V, Sinyinza F, Lishimpi K, Nunn A, et al., 2006** [**11**] **.** This study evaluates the determinants of survival of HIV-infected children without ART in Zambia. Data from this study demonstrates that malnutrition and hospitalizations for respiratory or bacterial infections predict mortality independent of immunosuppression.

**Kekitiinwa** **A, Lee KJ, Walker AS, Maganda A, Doerholt K,** **et al., 2008** [**15**] **.** Few studies have directly compared responses to ART between children living in resource-rich and resource-poor settings. This study showed that irrespective of settings, overall immunological and virological responses to ART were similar.

## Abbreviations

ART: antiretroviral therapy
